# Antioxidant Therapy in Graves’ Orbitopathy

**DOI:** 10.3389/fendo.2020.608733

**Published:** 2020-12-14

**Authors:** Giulia Lanzolla, Claudio Marcocci, Michele Marinò

**Affiliations:** Department of Clinical and Experimental Medicine, Endocrinology Unit II, University of Pisa and University Hospital of Pisa, Pisa, Italy

**Keywords:** Graves’ orbitopathy (GO), oxidative stress, Quercetin, Enalapril, Vitamin C, N-acetyl-l-cysteine, melatonin, β-carotene

## Abstract

The balance of the cell redox state is a key point for the maintenance of cellular homeostasis. Increased reactive oxygen species (ROS) generation leads to oxidative damage of tissues, which is involved in the development of several diseases, including autoimmune diseases. Graves’ Orbitopathy (GO) is a disfiguring autoimmune-related condition associated with Graves’ Disease (GD). Patients with active, moderate-to-severe GO, are generally treated with high doses intravenous glucocorticoids (ivGCs) and/or orbital radiotherapy. On the contrary, up to recently, local ointments were the treatment most frequently offered to patients with mild GO, because the risks related to ivGCs does not justify the relatively poor benefits expected in mild GO. However, a medical treatment for these patients is heavily wanted, considering that GO can progress into more severe forms and also patients with mild GO complain with an impairment in their quality of life. Thus, based on the role of oxidative stress in the pathogenesis of GO, a therapy with antioxidant agents has been proposed and a number of studies have been performed, both *in vitro* and *in vivo*, which is reviewed here.

## Introduction

Graves’ Orbitopathy (GO) is an autoimmune, disfiguring disease observed in ~25%–30% of patients with Graves’ Disease (GD). More rarely, GO can be found in patients with hypothyroid autoimmune thyroiditis or in subjects with subclinical evidence of thyroid autoimmunity, but with no thyroid dysfunction, and the pathogenesis of these conditions is still a matter of debate (1, 2). Patients affected with moderate-to-severe and active GO are generally treated with high dose intravenous glucocorticoids (ivGCs), orbital radiotherapy, surgical procedures (3) as well as new medications, including Rituximab, an anti-CD20 monoclonal antibody (4), Teprotumumab, an anti-insulin-like growth factor-1 receptor (IGF1R) monoclonal antibody (5, 6), Tocilizumab, an interleukin 6 (IL-6) receptor antagonist (7) and mycophenolate (8). Fortunately, the majority of GO patients have a mild disease and are typically treated with local measures (3), because the risks carried by the above-mentioned therapies is not justified in view of the relatively poor benefits in mild GO, unless there is an important impairment of the quality of life of patients (9–11). Nevertheless, patients affected with mild GO still complain with a significant impairment in their quality of life, and although GO can improve spontaneously in up to 50% of them, it can worsen in ~15% of patients, thereby leading to the search for treatments that are suitable for these patients (2, 12). Oxidative stress plays an important role in the pathogenesis of GO, being involved in the production of pro-inflammatory cytokines and hyaluronic acid (HA), as well as in promoting proliferation of fibroblasts and their differentiation into adipocytes (13). Keeping in mind that an ideal treatment for mild GO should be effective, well tolerated and widely available, several antioxidant agents have been investigated as new possible approaches for the management of these patients. The role and efficacy of selenium have been well demonstrated, as extensively discussed in another review in the same issue of this journal. In addition to selenium, various antioxidant agents, namely pentoxifylline, nicotinamide, allopurinol, quercetin, enalapril, vitamin C, N-acetyl-L-cysteine and melatonin, have been proposed to play a potential therapeutic role in GO (14–18).

## Oxidative Stress in Graves’ Orbitopathy

**Figure 1:**
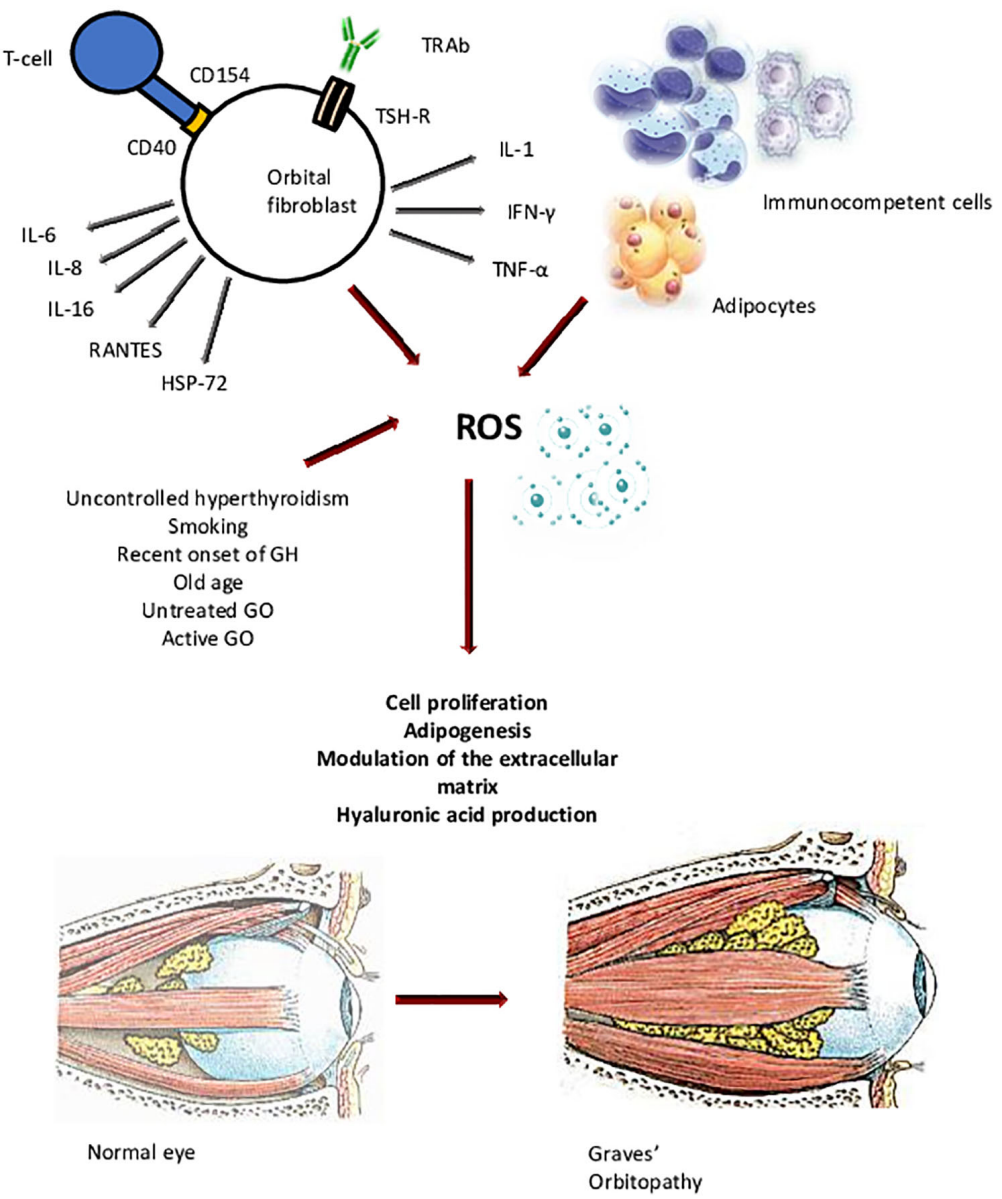
Orbital fibroblasts (OFs) are activated by both cellular and humoral immune responses against autoantigens, among which the thyrotropic hormone (TSH) receptor (TSH-R) is the main involved. The inflammatory process triggers the production of pro-inflammatory cytokines, growth factors, chemokines, and ROS, which contribute the increased proliferation of OFs, the differentiation of connective orbital cells into pre-adipocytes and then adipocytes, and the synthesis of great amounts of glycosaminoglycans (GAGs), especially hyaluronic acid. The resulting orbital remodeling is characterized by extraocular muscle enlargement and orbital fat expansion, which are ultimately responsible for the clinical manifestations of the disease. IL-6, interleukin-6; IL-8, interleukin-8; IL-16, interleukin-16; RANTES, regulated upon activation, normal T-cell expressed and presumably secreted; HSP-72, heat shock protein-72; IL-1, interleukin-1; IFN-γ, interferon γ; TNF-α, tumor necrosis factor α; GH, Graves’ hyperthyroidism; GO, Graves’ orbitopathy.

Oxidative stress is defined as the disruption of the balance between the production and the elimination of reactive oxygen species (ROS), which causes a remarkable damage of several cell components, namely proteins, lipids, membranes, and nucleic acids (19, 20). The process ultimately results in mitochondrial dysfunction and loss of enzymatic activity. The ultimate pathogenetic mechanisms of GO are still unclear, but the most popular hypothesis implies an interplay between cellular and humoral immunity against the thyrotropic hormone (TSH) receptor (TSH-R) and possibly other autoantigens expressed by thyrocytes and orbital fibroblasts (OFs) (2). Following recognition of these antigens by autoreactive T lymphocytes, they infiltrate fibroadipose orbital tissue, thereby triggering the production of pro-inflammatory cytokines, growth factors and chemokines. The inflammatory response and the consequent local cell damage cause a dysregulated production of ROS, with saturation of the antioxidant systems. The interplay between cellular and humoral immunity, as well as the uncontrolled release of inflammatory molecules and ROS, leads to the increased proliferation of OFs, the differentiation of connective orbital cells into pre-adipocytes and then adipocytes, and the synthesis of great amounts of glycosaminoglycans (GAGs), especially hyaluronic acid (HA). The resulting orbital remodeling is characterized by extraocular muscle enlargement and orbital fat expansion, which are ultimately responsible for the clinical manifestations of the disease (12, 21, 22). Among the various players involved in the pathogenesis of GO, oxidative stress is believed to have a major role. Tissue hypoxia, as well as ROS, are involved in the typical changes of fibroadipose orbital tissue and of the perimysium of extraocular muscles (23) (**Figure 1**). ROS, and the consequent antioxidant defense mobilization, are present at sites of inflammation in general and, in the case of GO, together with edema and tissue hypoxia, contribute the damage of orbital tissue. A role of oxidative stress in GO is supported by a number of basic studies suggesting that ROS promote the proliferation of OFs and the release of GAGs, the synthesis and secretion of cytokines, and the expression of other factors involved in the pathogenesis of GO, namely heat shock protein 72 (HSP-72), HLA-DR and ICAM-1 (14).

In this context, a number of studies, performed both *in vitro* and *in vivo*, investigated the effects of antioxidant agents in GO, including selenium, pentoxifylline, quercetin, enalapril, vitamin C, N-acetyl-L-cysteine and melatonin (14–18, 24, 25). Some of these studies have provided convincing or at least promising results, and in the case of selenium, have led to the clinical use of these antioxidant agents. In addition to the above mentioned compounds, statins (3-hydroxy-3-methylglutaryl-coenzyme reductase inhibitors), because of their anti-oxidative actions, have also been considered for GO treatment (26).

### Oxidative Stress and Proliferation of Orbital Fibroblasts

Burch et al. performed a study on primary culture of OFs obtained from GO patients and control subjects, demonstrating that superoxide radicals induced an increased proliferation of OFs from GO patients compared with control fibroblasts (27). Moreover, preincubation of OFs with anti-thyroid drugs (ATD) and antioxidant agents as allopurinol and nicotinamide inhibited the proliferation induced by ROS (27).

The hypothesis that oxidative stress contributes GO pathogenesis was also suggested by studies performed by Tsai et al. and Hondur et al. (28, 29). They both showed that H_2_O_2_, a potent ROS-inducer, and superoxide dismutase (SOD), a natural antioxidant agent, were higher in GO then control OFs, whereas the ROS antagonist glutathione peroxidase (GPX) was reduced, with a reduced ratio between glutathione and oxidized glutathione, suggesting the presence of a remarkable state of oxidative stress (28–31).

### Glycosaminoglycan Production and Heat Shock Protein 72 Expression in Orbital fibroblasts

As mentioned above, the release of GAGs plays an important role in the pathogenesis of GO. The phenomenon is triggered by a number of cytokines, among which interleukin-1 (IL-1) is mostly involved (1, 2). Lu et al. reported that IL-1β promotes an increase in ROS release in OFs from patients with GO (32). SOD and catalase, another ROS antagonist, to some extent reduced the secretion of GAGs induced by IL-1β, suggesting that ROS may be involved in the IL-1β-dependent release of GAGs. In addition, pentoxifylline, which has antioxidant properties, was found to be able to inhibit GAG accumulation in GO OFs (33). HSP-72 is involved in antigen recognition and T-lymphocyte activation in orbital tissue, and its expression in GO fibroblasts is promoted by H_2_O_2 (_ 34_),_. The effects of H_2_O_2_ were reduced by anti-oxidative agents or ATD (35), suggesting that ROS play a role in the aberrant expression of HSP-72 in OFs of GO.

### Clinical Evidence of a Role of Oxidative Stress in Graves’ Orbitopathy

In addition to basic studies, the importance of oxidative stress in GO is supported by clinical investigations. Cigarette smoking is the most important environmental risk factors for GO. Thus, cigarette smoke enhances the *in vitro* generation of ROS and reduces the antioxidant machinery (36). Patients with a recent onset Graves’ hyperthyroidism (GH) have higher plasma levels of SOD and catalase than control subjects, regardless of the presence of GO (37). No differences in serum GPX and thioredoxin reductase (TRs), another ROS antagonist, were observed. However, the normalization of oxidative markers following restoration of euthyroidism was observed only in patients without GO, suggesting that orbital inflammation contributes the increased serum markers of oxidative stress (38–40). Moreover, higher urinary levels of 8-hydroxy-2’-deoxyguanosine (8-OHdG), a marker of oxidative DNA damage, were found in patients with active GO (29, 31). Interestingly, urinary 8-OHdG was higher in smokers and in patients with GO relapse, and oral glucocorticoid (GC) administration led to a significant decrease of 8-OHdG levels, which was also associated with a reduction of GO activity and severity. These data were confirmed by a subsequent study performed in a larger cohort of GO patients, where there was a significant positive correlation between the GO Clinical Activity Score (CAS) and urinary levels of 8-OHdG. The relationship between oxidative stress and GC has also been studied (30) Akarsu et al. reported a significant reduction in the serum levels of malondealdyde (MAD), a marker of oxidative stress, following GC administration in GO patients (41). Similar findings were obtained by Abalovich et al. (38) and Bednarek et al. (39), in line with an involvement of oxidative stress in the pathogenesis of GO.

## Antioxidant Agents in Graves’ Orbitopathy

According to the guidelines of the European Thyroid Association and of the European Group on Graves’ Orbitopathy (EUGOGO), a number of therapies can be offered to patients with moderate-to-severe and active GO. Even though mild GO often improve spontaneously (in ~50% of cases), it worsens in up to 15% of patients (3, 12) and, because of the disfiguring features of the disease and the impairment in the quality of life, also patients with mild GO ask for some sort of treatment, which has been searched for over the last decades. Keeping in mind that an ideal treatment for mild GO should be effective, well tolerated and widely available, antioxidant agents have been proposed as a reasonable approach. Selenium has been largely investigated and currently used in the clinical practice but will not be mentioned in this review article as it is treated largely elsewhere in this special issue. In addition to selenium, a protective role in GO has been suggested by studies on other antioxidant agents, as reported below and summarized in **Table 1**.

**Table 1:** Antioxidant agents in Graves’ orbitopathy (GO).

*Compound*	*Main finding*	*Methods*	*Results*	*Reference*
*Nicotinamide and Allopurinol*	Clinically significant improvement of GO	A pilot study including 22 consecutive patients with mild or moderately severe, active GO of recent onset (<6 months). They were treated with placebo or allopurinol (200 mg daily) plus nicotinamide (300 mg daily) for 3 months.	After 3 months, a significant improvement of GO was observed in 9 (82%) and 3 (27%) patients who respectively received antioxidant agents or placebo. The inflammation of soft tissue was the feature that responded more, whereas proptosis was little affected.	(42) Bouzas EA. et al.
*Pentoxifylline*	Inhibition of GAG release and fibroblast proliferation	Primary cultures of OFs from GO patients and control subjects were obtained. Cell proliferation and GAG production were measured after the addition of pentoxifylline (0.1–1,000 mg/L).	The exposure of OFs cultures to pentoxifylline resulted in a dose-dependent inhibition of fibroblast proliferation and GAG synthesis.	(33) Chang C.C et al.
	Clinically significant improvement of GO	A pilot study, neither randomized nor controlled, which involved 10 patients with active, mild or moderate-to-severe GO. Patients were treated with a daily infusion of pentoxifylline (200 mg/die) for 10 days, following which treatment was continued orally (1,800 to 1,200 mg/day) and stopped after 3 months.	At the end of treatment, a significant improvement of GO was observed in 8 of 10 patients. The beneficial effect was more evident on soft tissue inflammation, whereas diplopia and proptosis were less affected.	(43) Balazs C. et al.
	No improvement of GO	A prospective, multicenter, placebo-controlled clinical trial in which 159 patients with mild GO were randomized to receive sodium selenite (100 μg twice daily orally), pentoxifylline (600 mg twice daily orally) or placebo for 6 months.	Unlike selenium, no beneficial effects of pentoxifylline were observed	(44) Marcocci NEJM
*Quercetin*	Reduction of cell proliferation and HA release in GO fibroblasts	Primary cultures of OFs from GO patients and control subjects were obtained. Cell proliferation, cell necrosis, apoptosis and HA release were measured after incubation with medium without compounds, or containing: i) quercetin, ii) quercitrin, or iii) rutin. (at concentrations ranging between 1 and 150 μMCell).	Beginning at a 30 μM concentration, quercetin reduced cell proliferation and HA release, acting by the induction of necrosis and cell cycle blockade. No difference between GO and control fibroblasts was observed.	(16) Lisi S. et al.
*N-acetyl-cysteine, vitamin C and melatonine* .	Reduction of proliferation and release of cytokines in GO fibroblasts	Primary cultures of OFs from GO patients and control subjects. Oxidative stress was induced by H_2_O_2_. Cells were pretreated with N-acetylcysteine (100 and 200 μM) or vitamin C (250 and 500 μM). Cell proliferation, cell necrosis, apoptosis and HA release were measured	Treatment with H_2_O_2_ at low concentrations of H_2_O_2_ (3.125–25 μM) increased the survival of GO fibroblasts. 6.25 μM H_2_O_2_ led to a significant elevation of TGF β, IL-1 β and superoxide anion in GO fibroblasts, in GO, but not in control OFs. Pretreatment with N-acetylcysteine or vitamin C reversed the enhanced proliferation and the production of TGF-β, IL-1β and superoxide anion of GO fibroblasts in response to 6.25 μM H_2_O_2_.	(24) Tsai CC. et al.
	Reduction of cell proliferation and HA release in GO fibroblasts	GSSG, as a measure of oxidative stress, cell proliferation, HA, TNFα, IFNγ, and IL-1β were measured in primary cultures of GO and control OFs treated with H_2_O_2_ and incubated with N-acetyl-cysteine, vitamin C and melatonine	Oxidative stress was reduced by all of the three antioxidant agents. Vitamin C reduced proliferation in GO, but not in control fibroblasts. N-acetyl-l-cysteine reduced proliferation and IFNγ in GO, and HA and IL-1β in both GO and control fibroblasts. Melatonin reduced IL-1β and HA in GO and control fibroblasts, and IFNγ only in GO fibroblasts.	(17) Rotondo Dottore G. et al
*Enalapril* .	Reduction of cell proliferation and HA release in GO fibroblasts	Primary cultures of GO and control fibroblasts were treated with enalapril (2 or 5 mM, for 3 or 5 days) or with a control compound (lisinopril). Cell proliferation assays, lactate dehydrogenase release assays (as a measure of cell necrosis), apoptosis assays, and measurement of HA in the cell media were performed	Enalapril reduced OFs proliferation and HA release in the cell media in both GO and control fibroblasts. Because enalapril did not affect cell necrosis and apoptosis, the effects on proliferation probably reflected an inhibition of cell growth and/or a delay in cell cycle.	(15) Botta R. et al.
*Retinol*, *β-carotene*, *vitamin E*	Reduction of cell proliferation in GO fibroblasts	Primary cultures of GO and control fibroblasts. Oxidative stress was induced by treatment with H_2_O_2._ Cells were pre-incubated for 2 days at 37°C with complete medium without compounds, or with medium containing either one of the compounds investigated at various concentrations. GSSG, cell proliferation, HA, TNFα, IFNγ, and IL1β were measured	Retinol, β-carotene and vitamin E significantly reduced the release of GSSG and IL-1β induced by H_2_O_2_ in GO, but not in control fibroblasts. β-carotene reduced OFs proliferation in GO, but not in control fibroblasts, whereas retinol and vitamin E had no effect. Retinol reduced IFNγ in GO and control fibroblasts.	(16) Rotondo Dottore G. et al

OFs, orbital fibroblasts; GAG, glycosaminoglycans; HA, hyaluronic acid; H_2_O_2_, hydrogen peroxide; TGF β, transforming growth factor β; IL-1β, interleukin-1β, IFNγ, interferon γ; GSSG, Glutathione disulfide; TNFα, tumor necrosis factor α.

### Nicotinamide and Allopurinol

Allopurinol is a drug widely used in the management of hyperuricemia and gout, with powerful antioxidant activities, being an inhibitor of the enzyme xanthine oxidase (45). Vitamin nicotinamide is a largely studied nicotinamide adenine dinucleotide (NAD^+^) precursor, which is a coenzyme involved in a number of metabolic pathways and in the balance of cell redox state (46). The effects of nicotinamide are currently being investigated by some clinical trials (46). The potential role of allopurinol and nicotinamide in pathophysiologic conditions involving oxidative stress led to the investigation of their effects in GO. As mentioned above, Burch et al. reported that allopurinol and nicotinamide inhibit the proliferation of OFs induced by superoxide (27). This evidence was confirmed by a subsequent *in vitro* study performed in cultured OFs from patients with GO, incubated with interferon γ (IFN-γ) and tumor necrosis factor α (TNF-α) in the presence of nicotinamide (47). The authors reported an inhibitory effect of nicotinamide on cytokine production and OF proliferation. Moreover, nicotinamide was also able to reduce HSP-72 and HLA-DR as well as the expression of ICAM-1 expression, which is involved in the recruitment of T-lymphocytes to the orbit and enhances immune reactions and cytotoxicity (47–50). Bouzas et al. performed the first clinical, pilot study on antioxidants, which included 22 consecutive patients with mild or moderately severe GO treated with placebo or allopurinol (200 mg daily) plus nicotinamide (300 mg daily) for 3 months (42). All patients recruited had an active GO of recent onset (<6 months) and were euthyroid for at least 2 months. The response to treatment was evaluated by a complete ophthalmologic examination 1 and 3 months after the beginning of treatment. A significant improvement of GO was observed in 9 (82%) and 3 (27%) patients who respectively received antioxidant agents or placebo. The inflammation of soft tissue was the feature that responded more to the treatment, whereas proptosis was little affected. Clearly, further studies in larger series of patients are needed to confirm these promising results.

### Pentoxifylline

Pentoxifylline is an analog of the methylaxantine theobromine with anti-inflammatory and antioxidant properties (51). It is commonly used in the management of patients with vascular diseases (52). It has been demonstrated that pentoxifylline inhibits the synthesis of GAG and the proliferation of human skin fibroblasts (53). Similar results were reported in primary cultures of fibroblasts derived from patients with fibrotic diseases, including hypertrophic scar, scleroderma and keloid (54). In 1993 Chang et al. reported that treatment with pentoxifylline inhibits GAG release and fibroblast proliferation in primary cultures of OFs derived from patients with GO and control subjects, with no significant difference between them (33). Based on this evidence, some clinical studies investigated the potential beneficial effect of pentoxifylline in patients with GO. Balazs et al. performed a pilot study, neither randomized nor controlled, in 10 patients with GO selected for having contraindications to GCs (43). They were euthyroid for at least 4 weeks and they had a mild or moderate-to-severe GO. The patients were treated with a daily infusion of pentoxifylline (200 mg/die) for 10 days, following which treatment was continued orally (1800 to 1200 mg/day) and stopped after 3 months. At the end of treatment, a significant improvement of GO was observed in 8 of 10 patients. The beneficial effect was more evident on soft tissue inflammation whereas diplopia and proptosis were less affected by treatment. The randomized clinical trial performed by Marcocci et al. which opened for the clinical use of selenium in patients with GO, also investigated the effects of pentoxifylline. The authors showed that pentoxifylline was not associated with a significant improvement of the eye disease in terms of overall clinical outcome as well as the quality of life of patients (44).

### Quercetin

Quercetin is a member of the flavonoid family contained in vegetables and fruits, which assures an adequate intake with diet (16). Quercetin has anti-inflammatory, antioxidant, anti-viral activity, and is also able to promote apoptosis in tumor cells (55, 56). It has been reported that quercetin blocks the transforming factor-β (TGF β)/Smad signaling pathway, leading to the inhibition of the proliferation of scar-derived fibroblasts (57, 58). In addition, an antifibrotic activity in fibroblasts and adipocytes from GO patient has been reported, and quercetin seems also to be able to reduce the adipogenesis induced by cigarette smoke-extract in GO fibroblasts. Based on these evidences, a number of *in vitro* studies evaluated the possibility that quercetin may reduce the proliferation of OFs in GO. A study performed in primary cultures of OFs from GO patients and control subjects demonstrated that quercetin exerts an inhibitory effect on fibroblasts proliferation and HA release, with no difference between GO and control fibroblasts (16). The reduction of cell proliferation was likely due to a pro-necrotic, rather than to a pro-apoptotic, effect of quercetin. These findings suggest that quercetin may have a therapeutic role in GO, but *in vivo* studies are needed to confirm this hypothesis, also considering that quercetin seems to act through necrosis, which may worsen autoimmunity against orbital antigens due to antigen exposure to the immune system.

### Enalapril

Enalapril is a rather common anti-hypertensive medication, and it has been reported to inhibit fibroblasts proliferation and HA production in cheloid scars (59, 60). In view of these effects, a study was carried out using primary cultures of OFs from patients with GO and control subjects, which were treated with enalapril or with a control compound (lisinopril) (15). Cell proliferation, apoptosis, cell necrosis and HA release were measured. A significant reduction of OFs proliferation and HA release upon enalapril, but not lisinopril treatment, was observed in both GO and control OFs. Because enalapril did not cause an increase in necrosis or apoptosis, its inhibitory effects on proliferation were interpreted as the consequence of a direct action on cell growth and/or of a delay in cell cycle. No clinical studies are available on the use of this promising medication in GO.

### Vitamin C, N-Acetyl-l-Cysteine, and Melatonin

Additional antioxidant agents, namely Vitamin C, N-acetyl-l-cysteine and melatonin, were tested in another *in vitro* study performed in primary cultures of OFs from six GO patients and six control subjects (17). After treatment with H_2_O_2_ to induce oxidative stress, primary cultures were incubated with the various compounds. Glutathione disulfide (GSSG), as gage of oxidative stress, cell proliferation, HA, TNF-α, IFN-γ, and interleukin 1- β (IL1-β) were measured (17). H_2_O_2_ induced oxidative stress, as demonstrated by an increase in GSSG, and promoted cell proliferation and cytokine release, but did not affect HA. Vitamin C reduced proliferation in GO, but not in control fibroblasts. N-acetyl-l-cysteine reduced fibroblasts proliferation and release of IFN-γ, a cytokine involved in the pathogenesis of GO, in OFs from GO patients, and it was also able to inhibit HA and IL1-β secretion in both GO and control OFs. Melatonin reduced IL1-β and HA release in GO and control OFs, whereas its inhibitory effect on IFN-γ release was significant only in GO OFs (17). Overall, the results of this study support a beneficial role of Vitamin C and N-acetyl-L-cysteine, suggesting a possible clinical use of these compounds. Furthermore, this study showed, for the first time, a beneficial effect of melatonin in terms of cytokines and HA synthesis. However, based on the lack of an inhibitory effect on fibroblasts proliferation, melatonin does not seem to be a strong candidate for a clinical use.

### β-carotene, Retinol, and Vitamin E

Among other bio-available substances with antioxidant effects, β-carotene seems to be rather promising for a use in GO patients. In a study performed in our laboratory (18), again using primary cultures of OFs from GO patients and control subjects, cells were pre-incubated with medium containing β-carotene, retinol or Vitamin E, or with medium without compounds, following induction of oxidative stress with H_2_O_2_. As in previous studies (17), oxidative stress promoted fibroblast proliferation, but it did not affect HA release. All of the three substances were able to reduce oxidative stress in GO, but not in control OFs (18). Cell proliferation, HA synthesis, as well as of pro-inflammatory cytokines were measured. Cell proliferation was reduced only by β-carotene. No effects in terms of HA synthesis were observed, whereas all of the cytokines measured, namely IL-1β, IFN-γ and TNF-α, which were increased by H_2_O_2_, were reduced by β-carotene as well as by retinol and Vitamin E (18). We can speculate that Vitamin E and retinol might not have an effect on GO *in vivo*, because they do not affect cell proliferation. On the contrary, β-carotene displayed antiproliferative effects in addition to an antioxidant and anti-inflammatory action, making it the most suitable candidate, among the three compounds studied, for a clinical use. Clearly, clinical studies are needed.

## Conclusions

The balance of the cell redox state is a key point in cellular homeostasis. The disruption of the balance between oxidative and reductive activities in the cells leads to oxidative stress, by an increase in ROS production and a reduction of their scavenging. ROS interfere with intracellular reactions, thereby damaging various cellular components. Oxidative stress is involved in a number of diseases and seems to play an important role in GO. The management of patients with mild GO represents a challenge for endocrinologists and ophthalmologists. In addition to selenium, studies *in vitro* on the effect of other antioxidant agents provided, at least in part, interesting results which, unfortunately, have not been yet completely confirmed by clinical studies. Nicotinamide and allopurinol seem to be effective in reducing the inflammation of soft tissue but the data sustaining this conclusion were obtained in a rather small sample of GO patients affected with both moderate-to-severe and mild GO (42). The *in vitro* results on the use of pentoxifylline are promising, but clinical studies provided conflicting results (43, 44). Among the other compounds, because of the *in vitro* findings reported above, one can speculate that quercetin and β-carotene might be the most promising, but clinical trials are needed to confirm this hypothesis.

## Author Contributions

GL wrote the manuscript. CM and MM contributed to the conception of the manuscript and revised the paper critically. All authors contributed to the article and approved the submitted version.

## Conflict of Interest

The authors declare that the research was conducted in the absence of any commercial or financial relationships that could be construed as a potential conflict of interest.
